# Loss of TLR4 in mouse Müller cells inhibits both MyD88-dependent and –independent signaling

**DOI:** 10.1371/journal.pone.0190253

**Published:** 2017-12-29

**Authors:** Li Liu, Jena J. Steinle

**Affiliations:** 1 Department of Anatomy and Cell Biology, Wayne State University School of Medicine, Detroit, MI, United States of America; 2 Department of Ophthalmology, Wayne State University School of Medicine, Detroit, MI, United States of America; University of Florida, UNITED STATES

## Abstract

Müller cells are key to metabolic and ionic regulation in the retina. They also produce a number of inflammatory mediators and are significantly affected in diabetic retinopathy. To investigate the role of toll-like receptor 4 (TLR4) in retinal Müller cells, we crossed TLR4 floxed with PDGFRα-Cre mice to eliminate TLR4 in retinal Müller cells. We performed Western blotting and ELISA analyses to determine whether loss of TLR4 affected myeloid differentiation primary response protein (MyD88)-dependent or –independent signaling, leading to reduced levels of tumor necrosis factor alpha (TNFα) and interleukin 1 beta (IL1β) in whole retinal lysates from the TLR4 floxed and TLR4-PDGFRα-Cre mice. Data show that TLR4-PDGFRα-Cre mice have reduced levels of both the MyD88-dependent and -independent signaling pathways. These studies confirm successful development of a Müller cell-specific TLR4 knockout mouse colony. These mice have reduced MyD88-dependent and –independent signaling pathway proteins, as well as reduced TNFα and IL1β levels. These mice can be used to dissect TLR4 signaling in disorders affecting retinal Müller cells.

## Introduction

Müller cells are the principal glial cells of the retina [1]. These cells extend the whole thickness of the retina, serving to buffer the many metabolic and ionic processes of neurons [2]. Literature has shown that Müller cells can produce tumor necrosis factor alpha (TNFα) and interleukin 1 beta (IL1β) when stimulated by high glucose or other inflammatory mediators *in vitro* [3, 4]. Studies have suggested that toll-like receptors (TLR) 2,3,4,5 are expressed on retinal Müller cells [1], but less is known about their function.

Work in other retinal cells has shown that TLR4 in bone marrow derived cells is involved in the progression of diabetic retinopathy [5]. Work has also shown TLR4 is increased in the retina of streptozotocin-treated diabetic rats [6]. TLR4 mediated the lipopolysaccharide (LPS)-induced preconditioning effects on multiple retina types through activation of retinal microglia [7]; however Müller cells were not specifically investigated in this study. Despite the relative paucity of information on TLR4 in the retina, work from other organ systems has provided a plethora of knowledge on TLR4 signaling. TLR activation can lead to a number of cardiovascular disorders, including artherosclerosis, cardiac dysfunction in sepsis, and congestive heart failure [8]. While most TLRs have specific ligands for activation, TLR4 may also activated by other danger signals, such as high glucose, either directly or indirectly. Work in retinal Müller cells and endothelial cells grown in high glucose showed that β-adrenergic receptor stimulation reduced TLR4 signaling [9]. Similarly, knockout of TLR4 in the diabetic retina attenuated TLR4 signaling [10]. A myeloid differentiation primary response protein (MyD88) chimera showed that TLR4 signaling was involved in retinal endothelial cell apoptosis [11]. In contrast, TIR domain-containing adaptor inducing IFN-β (TRIF) mediated apoptosis of bacteria-infected macrophages, with no response through MyD88-dependent signaling [12]. TRIF has also been shown to be key in the MyD88-independent signaling for TLR4 in TRIF-deficient macrophages [13]. Thus, TLR4 can signal via the MyD88-dependent or –independent pathways based upon cell specific responses.

For this study, we developed and characterized TLR4-Müller cell-specific conditional knockout mice. We used these mice to investigate whether loss of TLR4 in Müller cells affected MyD88-dependent or MyD88-independent signaling in retinal Müller cells.

## Methods

### Mice

All animal procedures meet the Association for Research in Vision and Ophthalmology requirements and were approved by the Institutional Animal Care and Use Committee of Wayne State University (A-08-07-15) and conform to NIH guidelines. The TLR4 floxed mice (B6(Cg)-Tlr4^tm1.1Karp^/J mice) and PDGFRα-Cre (C57BL/6-Tg(Pdgfra-cre)1Clc/J) mice were purchased from Jackson Laboratories. After 2 generations, the TLR4 floxed mice were bred with the TLR4-PDGFRα-Cre mice to generate conditional knockout mice in which TLR4 is eliminated in Müller cells. At 3 months of age, TLR4 floxed and TLR4-PDGFRα-Cre mice were used for these experiments. If we did not have successful knockout using the TLR4-PDGFRα-Cre, these littermates were grouped with the TLR4 floxed mice. Euthanasia was performed with CO_2_ followed by cervical dislocation.

### Genotyping

Genomic DNA was extracted from ear punch samples from 2-week-old mice. Ear punches were digested with one step tail DNA extraction buffer (100mM Tris, 5mM EDTA, 200mM NaCL, 1% Triton) plus proteinase K (10mg/ml) at 55°C overnight, followed by enzyme heat-inactivation at 85°C for 45 min. Primer pairs used to screen the TLR4 conditional knock out mice were as follows: TLR4: 5’->3’ mutant forward: TGA CCA CCC ATA TTG CCT ATA C and 5’->3’ mutant reverse: TGA TGG TGT GAG CAG GAG AG. PDGFRα cre 5’->3’ forward: GCG GTC TGG CAG TAA AAA CTA TC and reverse: GTG AAA CAG CAT TGC TGT CAC TT. The PCR reaction was done using KAPA2G HotStar Genotyping PCR Mix (KK5621, KAPA Biosystems). PCR reaction was performed with following temperatures and times: denature: 95°C 3 min, 35 cycles at 95°C, 15 sec, 60°C 15 sec, 72°C sec/kb, and a final extension at 72°C 1 min.

### Immunohistochemistry for TLR4

Three-month-old male and female TLR4 floxed and TLR4 PDGFRα-Cre mice (3 in each group) were euthanized by CO_2_, followed by cervical dislocation. LPS (0.5mg/kg) was used to activate the Müller cells 48 hours before sacrifice of the mice for immunohistochemistry experiments only. After confirmation of death, the eyes were removed and placed into in 4% paraformaldehyde in PBS for 2 h. Whole globes were transferred into 0.1M PBS, and retinas were dissected out. Retinas were rinsed in PBS and placed into 5% normal goat serum for 2h at room temperature to block nonspecific staining, followed by incubation with mouse anti-TLR4 (1:500, Abcam, USA) and rabbit anti glutamine synthase antibodies (1:1000, Abcam, USA) for 2 days at 4°C. After rising in 0.3% Triton/PBS, retinas were incubated with secondary antibody donkey anti-mouse conjugated to Alexa Fluor 555 or goat anti-rabbit Alexa Fluor 488 (1:1000, Life Technologies) overnight at 4°C. Retinas were then rinsed in PBS and counter stained with DAPI. Samples were whole mounted and examined on a Leica Confocal microscope.

### Western blotting

Retinal lysates from untreated TLR4 floxed and TLR4 Cre-Lox mice were rinsed with cold PBS, collected in lysis buffer containing protease and phosphatase inhibitors, and scraped into tubes. Equal amounts of protein were separated on precast tris-glycine gels (Invitrogen, Carlsbad, CA), and then blotted onto a nitrocellulose membrane. After blocking in TBST (10mM Tris-HCl buffer, pH 8.0, 150 mM NaCl, 0.1% Tween 20) and 5% (w/v) BSA, the membrane was treated with appropriate primary antibodies followed by incubation with secondary antibodies labeled with horseradish peroxidase. Antigen-antibody complexes were detected by chemilluminescence reagent kit (Thermo Scientific, Pittsburgh, PA). Primary antibodies used were TLR4, MyD88, TRIF, TRAF6, IRAK1, and IRF3 (Abcam, Cambridge, MA) and beta actin (Santa Cruz, Santa Cruz, CA).

### ELISA

A TNFα ELISA (Fisher Scientific, Pittsburgh, PA) was used according to manufacturer’s instructions with the exception that sample exposure to primary antibody occurred for 24hrs. One hundred micrograms of protein were used for the TNFα ELISA to insure equal protein amounts in all wells. The IL-1β ELISA (R&D Systems, Minneapolis, MN) was completed on whole retinal lysates according to manufacturer’s instructions with the exception that 120ug protein was loaded into all wells, and the primary antibody incubated overnight.

### Statistical analyses

T-Tests were used to determine statistical significance between groups. P<0.05 was considered statistically significant.

## Results

### TLR4-PDGFRα-Cre mice have less co-localization of TLR4 with glutamine synthase in the retina

**Fig 1:**
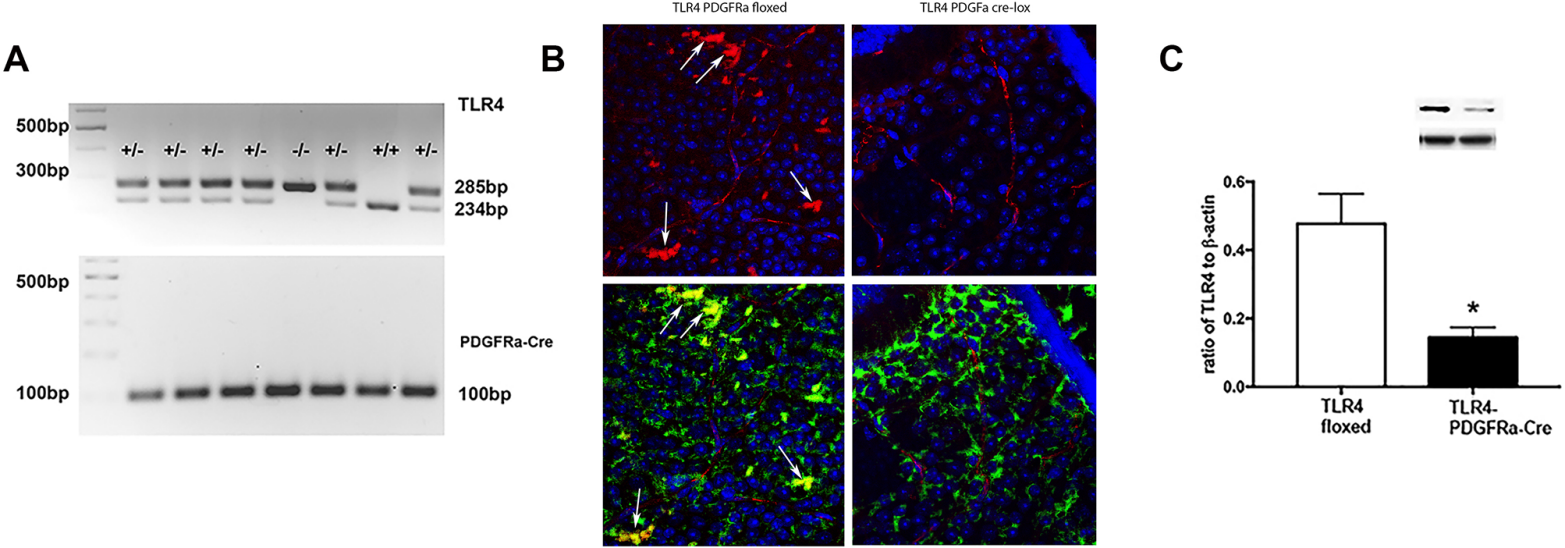
Panel A shows genotyping results for the TLR4 floxed and TLR4-PDGFRα-Cre mice. Panel B shows staining for TLR4 (red) and dapi (blue) in the TLR4 floxed and TLR4-PDGFRα-Cre mice in the top panels, while the lower panels show TLR4 (red) with glutamine synthase (green) to co-localize TLR4 in Müller cells. Panel C provides Western blot confirmation of TLR4 knockdown in whole retinal lysates. *P<0.05 vs. TLR4 floxed in Panel C. N = 5.

We first needed to characterize the mice used for these studies. We performed genotyping experiments to determine which mice were TLR4 floxed versus TLR4- PDGFRα-Cre mice (Fig 1A). We then performed immunofluorescence for TLR4 (red) in top panels to show TLR4 localization in the retina of the TLR4 floxed (left) and TLR4-PDGFRα-Cre mice (top right, Fig 1B). The lower panels show a co-localization of TLR4 (red) with glutamine synthase (green) to demonstrate reduced TLR4 levels in the Müller cells in the retina (Fig 1B, lower panels). We confirmed the staining results by Western blot on whole retinal lysates showing a significant reduction (P<0.05 vs. TLR4 floxed) in TLR4 levels in the TLR4-PDGFRα-Cre mice compared to TLR4 floxed mice (Fig 1C).

### Loss of TLR4 reduced MyD88-dependent signaling

**Fig 2:**
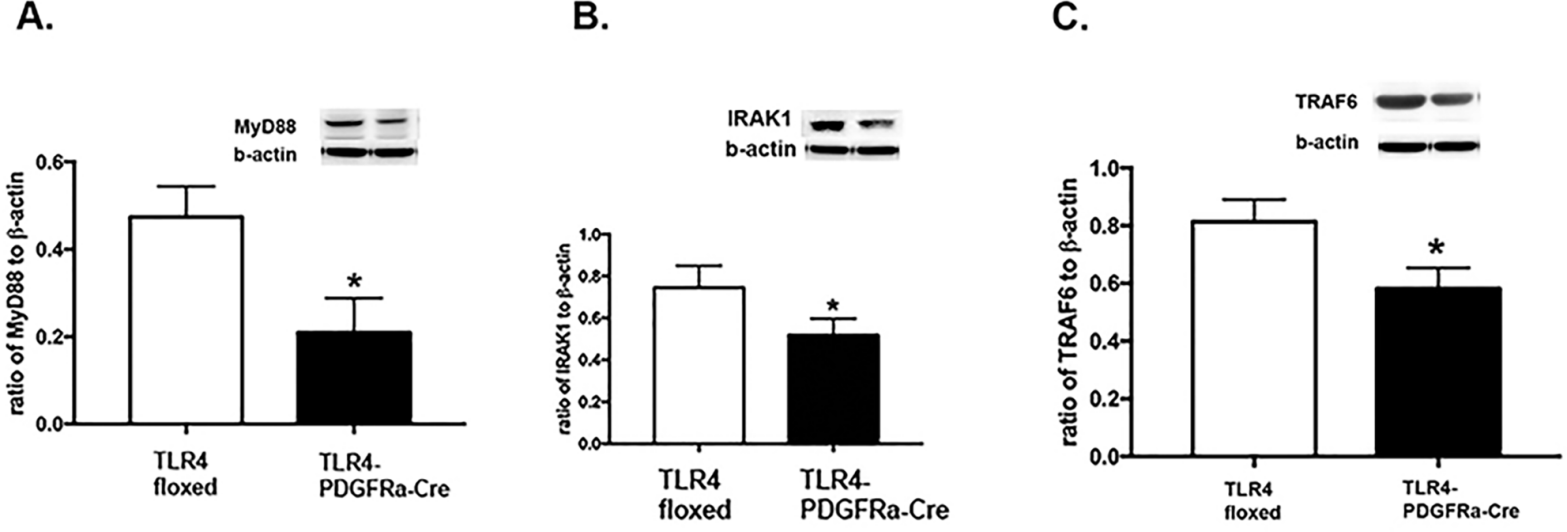
**Western blot results for MyD88 (A), IRAK1 (B) and TRAF6 (C) in whole retinal lysates from TLR4 floxed and TLR4-PDGFRα-Cre mice**. *P<0.05 vs. floxed. N = 5 for all groups.

TLR4 actions can be mediated by MyD88-dependent or –independent signaling [11, 12]. We measured protein levels of MyD88 (A), interleukin 1 receptor associated kinase 1 (IRAK1, B), and tumor necrosis factor receptor-associated factor 6 (TRAF6, C) in whole retinal lysates of the TLR4 floxed and TLR4-PDGFRα-Cre mice (P<0.05 vs TLR4 floxed for all 3 panels, Fig 2). Data show that MyD88-dependent signaling is reduced in retinal Müller cells after genetic deletion of TLR4.

### TLR4-PDGFRα-Cre mice have reduced TNFα and IL1β levels

**Fig 3:**
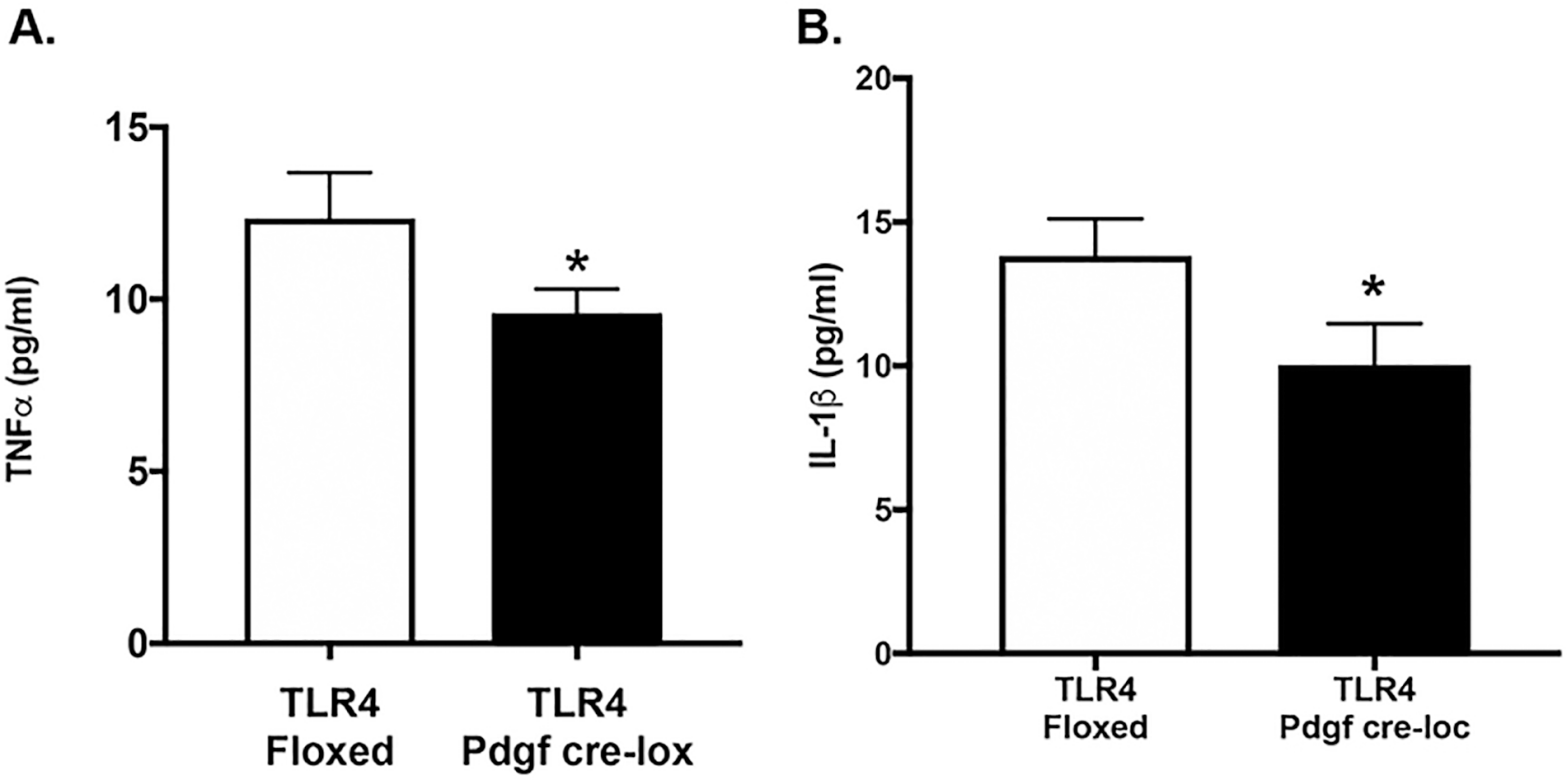
**ELISA levels of TNFα (A) and IL1β (B) in whole retinal lysates from TLR4 floxed and TLR4-PDGFRα-Cre mice**. *P<0.05 vs. floxed. N = 5 for all groups.

Since activation of the TLR4-MyD88 pathway activates a number of pro-inflammatory pathways, we wanted to measure 2 key proteins, which can be activated in retinal Müller cells, TNFα and IL1β [14, 15]. Fig 3A and 3B shows that loss of TLR4 significantly (P<0.05 vs. TLR4 floxed) reduced TNFα and IL1β levels, respectively, in whole retinal lysates from TLR4-PDGFRα-Cre mice.

### Loss of TLR4 in Müller cells also reduced MyD88-independent signaling pathways

**Fig 4:**
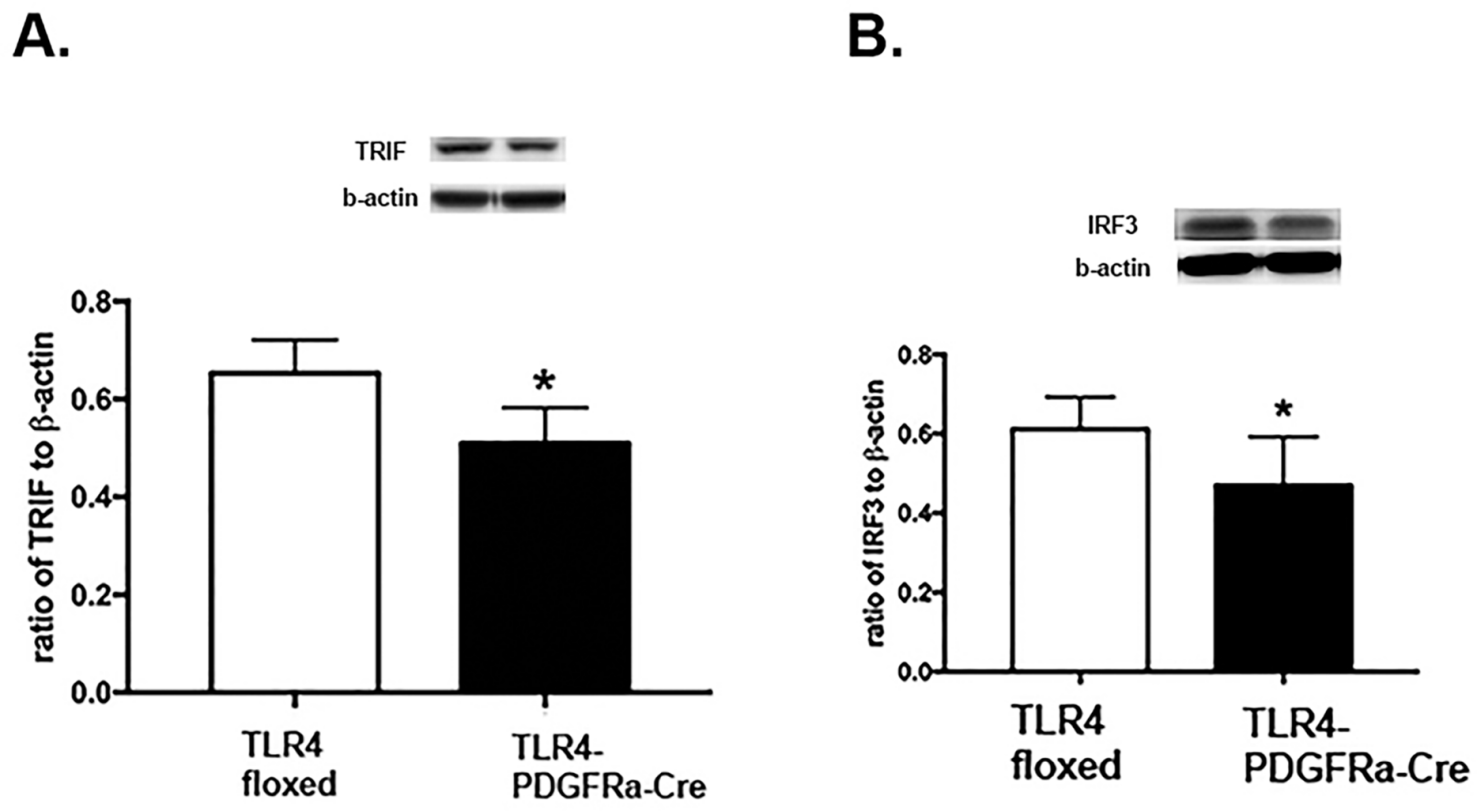
**Western blot results for TRIF (A) and IRF3 (B) in whole retinal lysates from TLR4 floxed and TLR4-PDGFRα-Cre mice**. *P<0.05 vs. floxed. N = 5 for all groups.

To investigate whether MyD88-independent signaling was also altered by the loss of TLR4 in retinal Müller cells, we measured protein levels of TRIF and interferon regulator factor 3 (IRF3) in whole retinal lysates. Fig 4 shows that loss of TLR4 in the TLR4-PDGFRα-Cre mice led to reduced TRIF and IRF3 levels in Müller cells, demonstrating that loss of TLR4 repressed MyD88-independent signaling.

## Discussion

The first goal of this study was to develop and confirm successful knockout of TLR4 in retinal Müller cells. Fig 1A and 1B confirm by gene expression and co-localization that TLR4 is eliminated in retinal Müller cells. Western blot of whole retinal lysates also showed a significant reduction in TLR4 levels in the entire retina from the TLR-4-PDGFRa-Cre mice when compared to the TLR4 floxed mice. We have previously reported altered vascular permeability and insulin signaling in endothelial cell specific TLR4 knockout mice [16, 17]. The current study was focused on Müller cells, since both endothelial and Müller cells are key cell types affected in diabetic retinopathy. We used PDGFRα Cre mice to breed with the TLR4 floxed miced to eliminate TLR4 in Müller cells. According to the Jackson laboratory website, “Hemizygous Pdgfra-cre mice are viable and fertile, with *cre* expression directed to retinal Muller glial cells by the mouse *Pdgfra* (platelet derived growth factor receptor, alpha polypeptide) promoter. Expression is predominantly in the cell bodies of the inner nuclear layer (INL) of the retina, but some expression may be observed in the outer nuclear layer (ONL) and in the ganglion cell layer (GCL). The donating investigator indicates that although not examined, *cre* may also be active in many types of central nervous system glial cells. When bred with mice containing a loxP-flanked sequence of interest, Cre-mediated recombination will result in deletion of the floxed sequence(s) in the offspring.” Using the information provided by Jackson laboratories and our gene and protein expression data suggest successful reduction of Müller cell TLR4 expression in the TLR4-PDGFRα-Cre mice.

Previous studies have shown that both endothelial cells and Müller cells have increased inflammatory mediator production in response to high glucose or inflammatory stimuli [3, 4]. Our findings of reduced inflammatory mediators in the Müller cell specific knockout mice are in agreement with other studies in diabetic rats [6] or diabetic human monocytes [18]. Whole animal knockout of TLR4 also showed reduced inflammatory mediator levels in diabetic mice [10]. Thus, our findings of reduced TNFα and IL1β levels in the retinal lysates of the TLR4-PDGFRα-Cre mice agree with studies done in diabetic models, with the addition of reporting the changes in Müller cells for the first time, as long as PDGFRα-Cre is highly specific for Müller cells.

In addition to development of the mice, we also sought to determine whether loss of TLR4 in Müller cells affected the cellular machinery required for MyD88-dependent or –independent pathways or both. In most systems, TLR4 will be activated by a stimulus, such as LPS, leading to activation of the MyD88-dependent pathway, leading to activation of AP-1 or NFkB. In contrast, TLR4 can also activate the MyD88-independent pathway, leading to IRF3 activation [8]. We have previously reported that β-adrenergic receptor activation reduced both MyD88-dependent and –independent pathways in retinal endothelial cells grown in high glucose [9]. In contrast, work in bacteria-infected macrophages regulated apoptosis through TRIF, but not MyD88-dependent pathways [12]. Thus, there appears to be cell-type specific responses to TLR4 activation. Some of these differences may be related to the quantity of Müller cells in the whole retinal lysates. Müller cells represent a small population of retinal cell types.

Now that we have characterized these mice and shown TLR4 signaling in the retinal Müller cells, we will extend these studies into work on ischemia/reperfusion (I/R) and diabetic retinopathy models. In both of these models, inflammatory pathways are activated. Use of the Müller cell specific conditional knockout mice will allow us to investigate the role of TLR4 in Müller cells in retinal I/R and diabetic retinopathy.

In conclusion, we had 2 primary goals for this study. We developed and confirmed successful knockdown of TLR4 in retinal Müller cells. We also found that loss of TLR4 caused a reduction in both the MyD88-dependent and –independent signaling in whole retinal lysates from Müller cell specific TLR4 knockout out mice.
